# Clinical diagnosis and treatment of 37 cases of gallbladder neuroendocrine carcinoma

**DOI:** 10.1186/s12957-024-03436-z

**Published:** 2024-06-14

**Authors:** Feng Liu, Wentao Miao, Jiang Nan, Zhiyong Shi, Anhong Zhang, Yunfeng Bo, Jun Xu

**Affiliations:** 1 First Clinical Medical School, Shanxi Medical University, Taiyuan, 030001 China; 2 Department of Head and Neck Surgery, Shanxi Provincial Cancer Hospital, Shanxi Hospital Cancer Hospital of Chinese Academy of Medical Sciences, Taiyuan, 030001 China; 3 Department of Plastic Surgery, Taiyuan Maternity and Child Health Care Hospital, Taiyuan Children’s Hospital, Taiyuan, 030001 China; 4 Department of Hepatobiliary and Pancreatic Surgery, Liver Transplantation Center, The First Hospital of Shanxi Medical University, Taiyuan, 030001 China; 5 Department of Pathology, Shanxi Hospital Affiliated to Cancer Hospital, Shanxi Province Cancer Hospital, Chinese Academy of Medical Sciences, Taiyuan, 030001 China

**Keywords:** Clinical features, Gallbladder neuroendocrine carcinoma, Survival time, Surgical treatment

## Abstract

**Objective:**

This study aims to investigate the clinical and pathological characteristics, treatment approaches, and prognosis of gallbladder neuroendocrine carcinoma (GB-NEC).

**Methods:**

Retrospective analysis was conducted on the clinical data of 37 patients with GB-NEC admitted to Shanxi Cancer Hospital from January 2010 to June 2023. The study included an examination of their general information, treatment regimens, and overall prognosis.

**Results:**

Twelve cases, either due to distant metastasis or other reasons, did not undergo surgical treatment and received palliative chemotherapy (Group 1). Two cases underwent simple cholecystectomy (Group 2); four patients underwent palliative tumor resection surgery (Group 3), and nineteen patients underwent radical resection surgery (Group 4). Among the 37 GB-NEC patients, the average pre-surgery CA19-9 level was 113.29 ± 138.45 U/mL, and the median overall survival time was 19 months (range 7.89–30.11 months). Of these, 28 cases (75.7%) received systemic treatment, 25 cases (67.6%) underwent surgical intervention, and 16 cases (64.0%) received postoperative adjuvant treatment, including combined radiochemotherapy or chemotherapy alone. The median overall survival time was 4 months (0.61–7.40 months) for Group 1 (*n* = 12), 8 months for Group 2 (*n* = 2), 21 months (14.67–43.33 months) for Group 3 (*n* = 4), and 19 months (range 7.89–30.11 months) for Group 4 (*n* = 19). A significant difference in median overall survival time was observed between Group 1 and Group 4 (*P* = 0.004).

**Conclusion:**

Surgery remains the primary treatment for GB-NEC, with radical resection potentially offering greater benefits to patient survival compared to other therapeutic options. Postoperative adjuvant therapy has the potential to extend patient survival, although the overall prognosis remains challenging.

## Introduction

Gallbladder carcinoma is a seldom encountered malignant tumor in clinical settings, with the potential to manifest in the fundus, body, neck, or bile duct of the gallbladder. Due to its atypical early symptoms and high malignancy, the overall therapeutic efficacy is limited, resulting in an unfavorable prognosis [1]. Neuroendocrine tumors (NENs) arise from dispersed neuroendocrine cells and peptidergic neurons throughout the body, predominantly occurring in the gastrointestinal and respiratory systems, displaying intricate heterogeneity [2–4]. Gallbladder neuroendocrine carcinoma (GB-NEC) is relatively uncommon in clinical practice, representing only 0.5-2% of all gallbladder tumors [5–7]. It is characterized by robust invasiveness, a high recurrence rate, and an unfavorable clinical prognosis [7, 8].

GB-NEC exhibits a low incidence and is primarily documented in isolated cases or small series. Controversies persist regarding the clinical characteristics and treatment modalities for this disease, with a notable absence of standardized diagnostic and treatment protocols. Surgical intervention plays a pivotal role in the comprehensive management of GB-NEC, yielding improved therapeutic outcomes in select patients. Consequently, this study retrospectively examined the clinical and pathological data of 37 GB-NEC patients, scrutinizing their clinical and pathological features, treatment modalities, and prognosis. The aim is to furnish clinical evidence and insights to enhance the overall therapeutic outcomes for this condition.

## Materials and methods

### Clinical data

We gathered clinical data from 37 patients diagnosed with gallbladder neuroendocrine carcinoma (GB-NEC), who were admitted to the General Surgery Department of Shanxi Cancer Hospital between January 2010 and June 2023. The patients received their GB-NEC diagnosis either at our hospital or elsewhere, and all subsequent treatments were conducted at our institution, with comprehensive follow-up information. Table 1 provides a summary of the clinical and pathological data for these 37 GB-NEC patients. This study has received approval from our institution’s ethics committee (Ethics number: KY2023115), and informed consent has been duly obtained from both the patients and their families.

**Table 1:** Clinical and pathological characteristics of 37 patients with GB-NEC

n = 37	
Age (years, X ± S)	60.35 ± 11.29
Gender (male:female)	12:25
Symptoms (n, %)	
Abdominal pain	28 (75.7)
Jaundice	5 (13.5)
Anorexia, emaciation	5 (13.5)
Others	4 (10.8)
Preoperative CA19-9 (U/mL, X ± S)	113.29 ± 138.45
Preoperative CEA (ng/mL, X ± S)	10.14 ± 23.74
Biliary tract disease (n, %)	
Cholecystolithiasis	16 (43.2)
Gallbladder polyps	3 (8.1)
Gallbladder adenomyomatosis	2 (5.4)
Choledochal cyst	1 (2.7)
Tumor location (n, %)	
Bottom	14 (37.9)
Neck	10 (27.0)
Body	9 (24.3)
NA	4 (11.8)
AJCC staging (n, %)	
Stage I	1 (2.7)
Stage II	5 (13.5)
Stage III	7 (18.9)
Stage IV	24 (64.9)
Treatment (n, %)	
Operation	25 (67.6)
Chemotherapy only	12 (32.4)
Status at last follow-up (n, %)	
Neoplastic death	28 (75.7)
Non-neoplastic death	1 (2.7)
With tumor survival	5 (13.5)
Disease-free survival	3 (8.1)

Note: NA = Not available

### Preoperative auxiliary examination and treatment regimens development

All patients underwent a preoperative assessment that included a complete blood count, liver and renal function tests, serum tumor marker detection, chest CT, abdominal ultrasound, enhanced CT and 18 F-FDG PET/CT to comprehensively evaluate the tumor situation. Treatment plans were determined and executed through discussions within the Multidisciplinary Team (MDT) with the informed consent of patients and their families. Before surgery, imageological examinations assessed the size and depth of the tumor, infiltration of adjacent organs, and the presence of distant metastasis. If no distant metastasis was identified, and the tumor was deemed removable, radical surgery was performed. The specific surgical procedure for tumor resection adhered to the guidelines for gallbladder carcinoma, and the extent of resection was decided by the surgeons based on intraoperative conditions. Factors considered included local tumor infiltration, lymph node metastasis, and the overall health status of the patient. In cases where distant metastasis was observed preoperatively, palliative anti-tumor treatment was administered as per the specific circumstances.

### Treatment methods

**Fig. 1:**
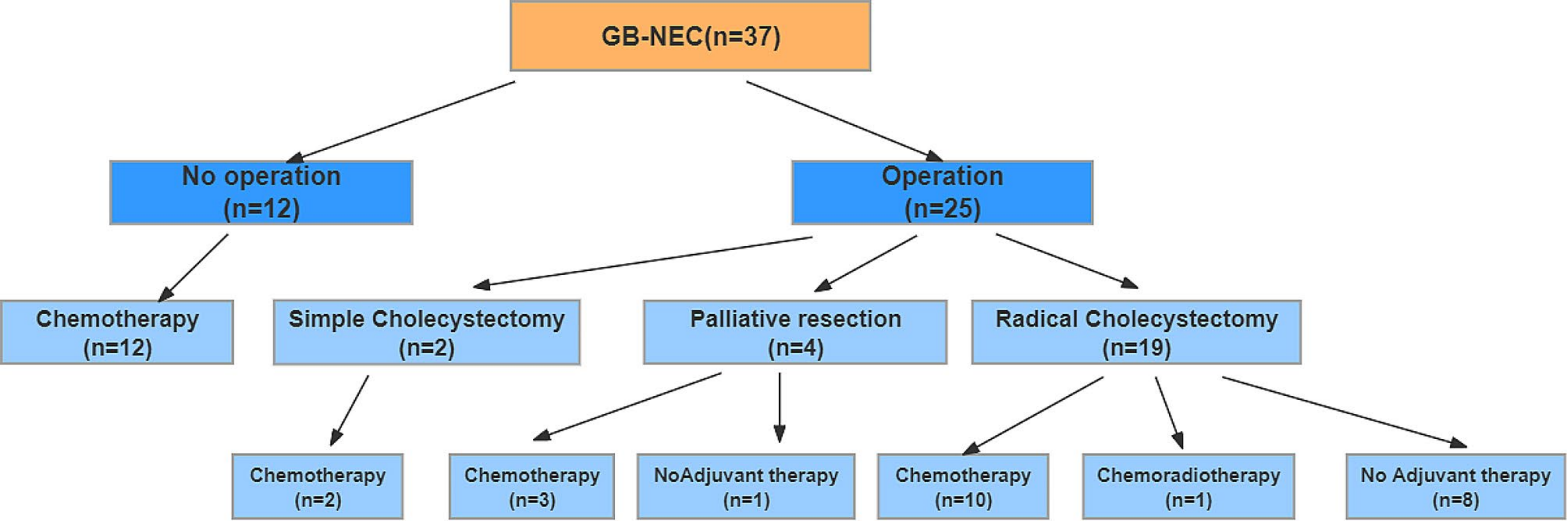
Treatment in patients diagnosed with GB-NEC

The specific treatment details for the 37 cases of GB-NEC patients are elucidated in Fig. 1. Among these individuals, 12 patients refrained from surgery and solely underwent palliative chemotherapy, as distant metastasis or other reasons(unwilling to take the risks of surgery)were confirmed. Two patients underwent simple gallbladder resection, and after postoperative pathology confirmed NEC, no further supplementary surgery was performed. Instead, they received EP regimen chemotherapy. Two patients presented with jaundice and underwent palliative jaundice-reducing surgery. Of these, one patient received EP regimen chemotherapy postoperatively, while the other did not undergo any adjuvant treatment. Two patients with extensive tumor invasion underwent palliative tumor-reducing surgery, followed by 3 cycles of GP (capecitabine + oxaliplatin) chemotherapy postoperatively. Nineteen patients underwent radical resection, among whom one received 4 cycles of neoadjuvant chemotherapy with the EP regimen, ten received adjuvant chemotherapy, one underwent combined radiochemotherapy, and eight did not receive any adjuvant treatment.

### Follow-up

In the initial 2 years post-surgery, patients attended outpatient follow-up appointments every 3 months. Subsequently, starting from the third year, follow-up appointments were scheduled every 6 months. For those who did not visit our outpatient facility for follow-up, telephone follow-up was implemented. Re-examinations encompassed both imageological and laboratory examinations. Throughout the follow-up period, disease recurrence was ascertained through either imageological or histopathological examinations. A dedicated research nurse conducted monthly telephone follow-ups to ascertain the survival status of patients. Overall survival was defined as the duration from the date of surgery or needle biopsy to death for any reason or until the last follow-up in December 2023. Disease-free survival was defined as the time from the date of radical surgery to the occurrence of tumor recurrence or metastasis or until the last follow-up date in December 2023.

### Statistical analysis

All statistical analyses were conducted using SPSS 25.0 and R 4.1.0 software. The measurement data, conforming to a normal distribution, were expressed as the mean ± standard deviation. Disease-free survival and overall survival were assessed utilizing the Kaplan-Meier method, with intergroup comparisons performed through the log-rank test. Statistical significance was established at *P* < 0.05.

## Results

### Basic information on patients with GB-NEC (37 cases)

Table 1 summarizes the key characteristics of the 37 patients diagnosed with GB-NEC. This patient cohort was predominantly composed of middle-aged and older women, with a mean age of 60.35 ± 11.29 years, and females constituted 67.6% (25 cases). Abdominal pain emerged as the most prevalent clinical manifestation, observed in 75.7% of cases (28 patients). The average preoperative CA19-9 and CEA levels were 113.29 ± 138.45 U/mL and 10.14 ± 23.74 ng/mL, respectively, exceeding normal ranges. Pathologic grading of all 37 cases was G3, Ki 67 30-90%. Biliary diseases were present in 59.5% of cases, with cholecystolithiasis being the most common (43.2% with 16 cases). Paraneoplastic syndrome-related symptoms, such as diarrhea, edema, flushing, or wheezing, were also noted. The median follow-up time was 13 months (range: 1-111 months), and the median overall survival time was 19 months (range: 7.89–30.11 months).

### Clinical, pathological, and prognosis status of patients with GB-NEC (Group 4)

Table 2 provides a comprehensive overview of the clinical and pathological characteristics of the 19 patients who underwent radical resection of the lesion. This cohort had a mean age of 60.00 ± 11.03 years, with 63.2% being female (12 cases). Abdominal pain was the predominant symptom, reported in 57.9% of cases (11 patients). Mean preoperative CA19-9 and CEA levels were 91.77 ± 105.61 U/mL and 18.34 ± 31.28 ng/mL, respectively, surpassing the normal range. Among the patients, 78.9% (15 cases) had biliary diseases, with cholecystolithiasis being the most prevalent (52.6% with 10 cases). Specific treatment and pathological data for these 19 patients are detailed in Tables 3 and 4, respectively.

**Table 2:** Clinical and pathological data of 19 patients undergoing radical surgical treatment

n = 19	
Age (years, X ± S)	60.00 ± 11.03
Gender (male:female)	7:12
Symptoms (n, %)	
Abdominal pain	11 (57.9)
Jaundice	3 (15.8)
Anorexia, emaciation	2 (10.5)
Others	3 (15.8)
Preoperative CA19-9 (U/mL, X ± S)	91.77 ± 105.61
Preoperative CEA (ng/mL, X ± S)	18.34 ± 31.28
Biliary tract disease (n, %)	
Cholecystolithiasis	10 (52.6)
Gallbladder polyps	3 (15.8)
Gallbladder adenomyomatosis	1 (5.3)
Choledochal cyst	1 (5.3)
Tumor location (n, %)	
Bottom	7 (36.8)
Neck	6 (31.6)
Body	4 (21.1)
NA	2 (10.5)
AJCC staging (n, %)	
Stage I	1 (5.3)
Stage II	4 (21.1)
Stage III	7 (36.8)
Stage IV	7 (36.8)
Adjuvant therapy (n, %)	
Chemotherapy	10 (52.6)
Chemoradiotherapy	1 (5.3)
No adjuvant therapy	8 (42.1)
Status at last follow-up (n, %)	
Neoplastic death	15 (78.9)
Non-neoplastic death	1 (5.3)
With tumor survival	1 (5.3)
Disease-free survival	2 (10.5)

Note: NA = Not available

**Table 3:** Specific treatment and prognosis status of 19 patients undergoing radical surgical treatment

No	Surgical method	Resection margin	Chemotherapy	Radiotherapy	Survival time (Months)	Status at last follow-up
1	Cho + LND + IVb, V	R0	EP6 cycles	No	7	Recurrence-free survival
2	Cho + LND + IVb, V	R0	No	No	23	Deceased
3	Cho + LND + RHP	R0	EP6 cycles	Yes	29	Deceased
4	Cho + LND + IVb, V	R0	No	No	3	Deceased
5	Cho + LND + IVb, V	R0	EP8 cycles	No	48	Survival with tumor
6	Cho + LND + RHP + BDR	R0	No	No	13	Deceased
7	Cho + LND + IVb, V	R0	EP6 cycles	No	8	Deceased
8	Cho + LND + IVb, V	R0	Neoadjuvant EP4 cycles + postoperative EP4 cycles	No	55	Deceased
9	Cho + LND + RHP	R0	No	No	4	Deceased
10	Cho + LND + IVb, V	R0	No	No	52	Deceased
11	Cho + LND + RHP	R0	No	No	11	Deceased
12	Cho + LND + IVb, V	R0	EP4 cycles	No	33	Deceased
13	Cho + LND + IVb, V + BDR	R0	EP6 cycles	No	54	Deceased
14	Cho + LND + IVb, V	R0	No	No	22	Deceased
15	Cho + LND + RHP	R0	EP6 cycles	No	46	Deceased
16	Cho + LND	R0	EP8 cycles	No	111	Recurrence-free survival
17	Cho + LND + IVb, V	R1	EP6 cycles	No	19	Deceased
18	Cho + LND + IVb, V	R0	No	No	4	Deceased
19	Cho + LND + RHP	R0	EP8 cycles	No	37	Deceased

Notes: BDR = bile duct resection

Cho = cholecystectomy

LND = lymph node dissection

IVb, V = subsegmentectomy of segments 4b, 5

RHP = right hemihepatectomy

**Table 4:** Pathological characteristics of 19 patients undergoing radical surgical treatment

No	Type of NEC	Adenocarcinoma component	Syn	CgA	CD56	CK	Ki-67
1	Small cell	None	+	+	+	+	90%
2	Small cell	None	+	+	+	+	80%
3	NA	None	+	+	+	+	90%
4	Large cell	None	+	-	+	+	70%
5	NA, 70%	30%	+	+	NP	+	80%
6	Large cell	None	+	+	+	+	50%
7	NA	None	+	-	+	+	80%
8	NA, 90%	10%	+	+	+	+	70%
9	NA, 80%	20%	+	-	NP	+	80%
10	NA, 70%	30%	+	+	+	+	90%
11	NA	None	+	-	+	+	90%
12	Small cell	None	+	-	+	+	80%
13	Large cell	None	+	+	+	+	80%
14	Small cell	None	+	-	+	+	50%
15	NA	None	+	-	+	+	90%
16	NA	None	+	+	+	+	90%
17	NA	None	+	+	+	+	80%
18	NA	None	+	+	NP	NP	90%
19	Small cell	None	+	+	NP	NP	50%

Note: NA = not available

NP = not performed

Remarkably, none of the 19 cases experienced inpatient or postoperative deaths within 30 days. The median follow-up time was 23 months (range: 3-111 months), with a median disease-free survival time of 8 months (range: 3.99–12.02 months) and a median overall survival time of 23 months (range: 8.45–37.55 months). Postoperative adjuvant therapy was administered to 11 cases, resulting in a median disease-free survival of 11 months (range: 8.05–13.95 months) and a median overall survival of 36 months (range: 14.26–57.74 months). In contrast, eight cases did not receive adjuvant therapy, with a median disease-free survival of 5 months (range: 3.15–6.85 months) and a median overall survival of 13 months (range: 7.87–18.13 months).

**Fig. 2:**
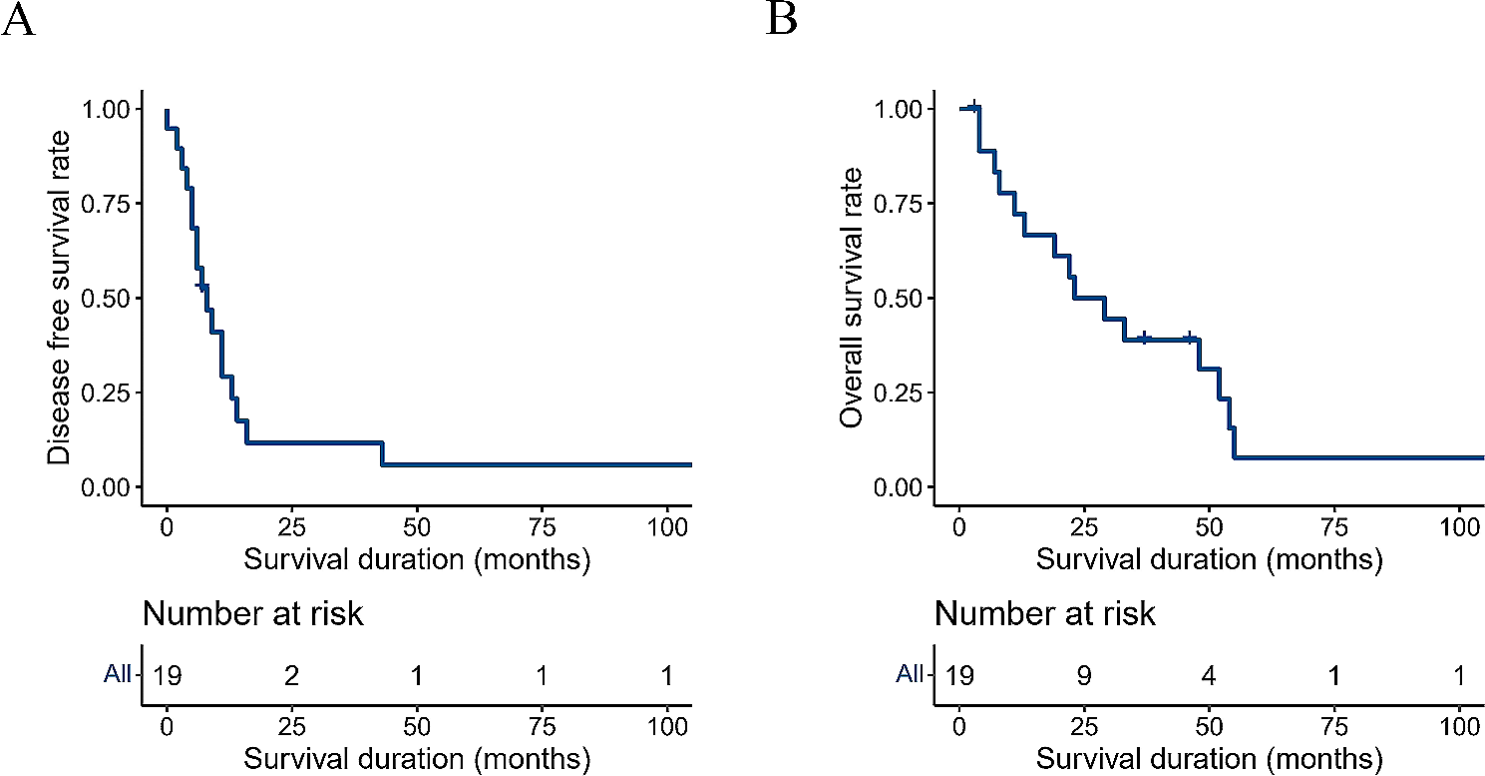
(**A**) Disease free survival rate of 19 patients undergoing radical resection surgery (Group 4); (**B**) Overall survival rate of 19 patients undergoing radical resection surgery (Group 4)

Among the 19 cases that underwent radical surgery, R0 resection was achieved in 94.7% (18 cases). Throughout the follow-up period, tumor recurrence or metastasis was identified in 83.3% (15 cases) through imaging or puncture biopsy. During the follow-up, 78.9% of patients (15 cases) succumbed to the disease, 5.3% (1 case) experienced non-tumor-related death at 4 months postoperatively due to acute myocardial infarction, 11.1% (2 cases) survived without tumors, and 5.3% (1 case) survived with tumors, as illustrated in Fig. 2A and B.

### Comparison analysis of treatment methods for GB-NEC

**Fig. 3:**
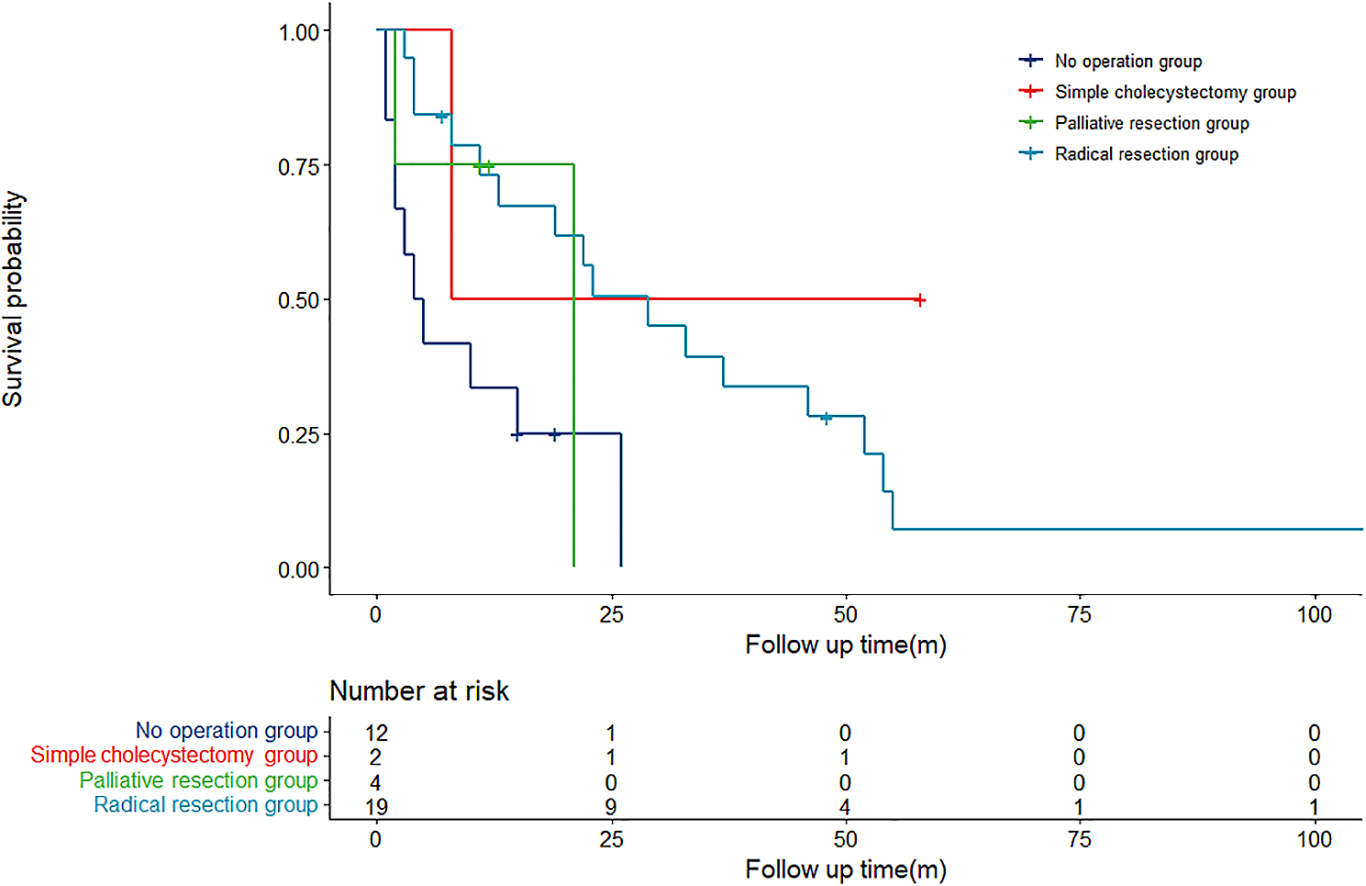
Overall survival rate of 37 GB-NEC patients (4 Groups)

Subgroup analysis by treatment methods was conducted, and the survival analysis of the 37 patients with GB-NEC is depicted in Fig. 3. The median overall survival was 4.0 months (0.61–7.40) for Group 1, 8.0 months for Group 2, 21.0 months (14.67–43.33) for Group 3, and 19.0 months (7.89–30.11) for Group 4. Notably, the median survival time of Group 4 was significantly longer than that of Group 1 (19.0 months vs. 4.0 months, *P* = 0.004). However, in a comparison with Group 3, Group 4 did not demonstrate superior survival outcomes (21.0 months vs. 19.0 months, *P* = 0.405). The comparison of overall survival in the four groups of patients is detailed in Table 5.

**Table 5:** Comparison of overall survival in 4 groups of patients

Group	1		2		3		4	
	X^2^	*P*	X^2^	*P*	X^2^	*P*	X^2^	*P*
1			1.190	0.275	0.836	0.361	8.071	0.004
2	1.190	0.275			0.076	0.782	0.776	0.378
3	0.836	0.361	0.076	0.782			0.693	0.405
4	8.071	0.004	0.776	0.378	0.693	0.405		

### Comparison analysis of overall survival in different stages of patients

**Fig. 4:**
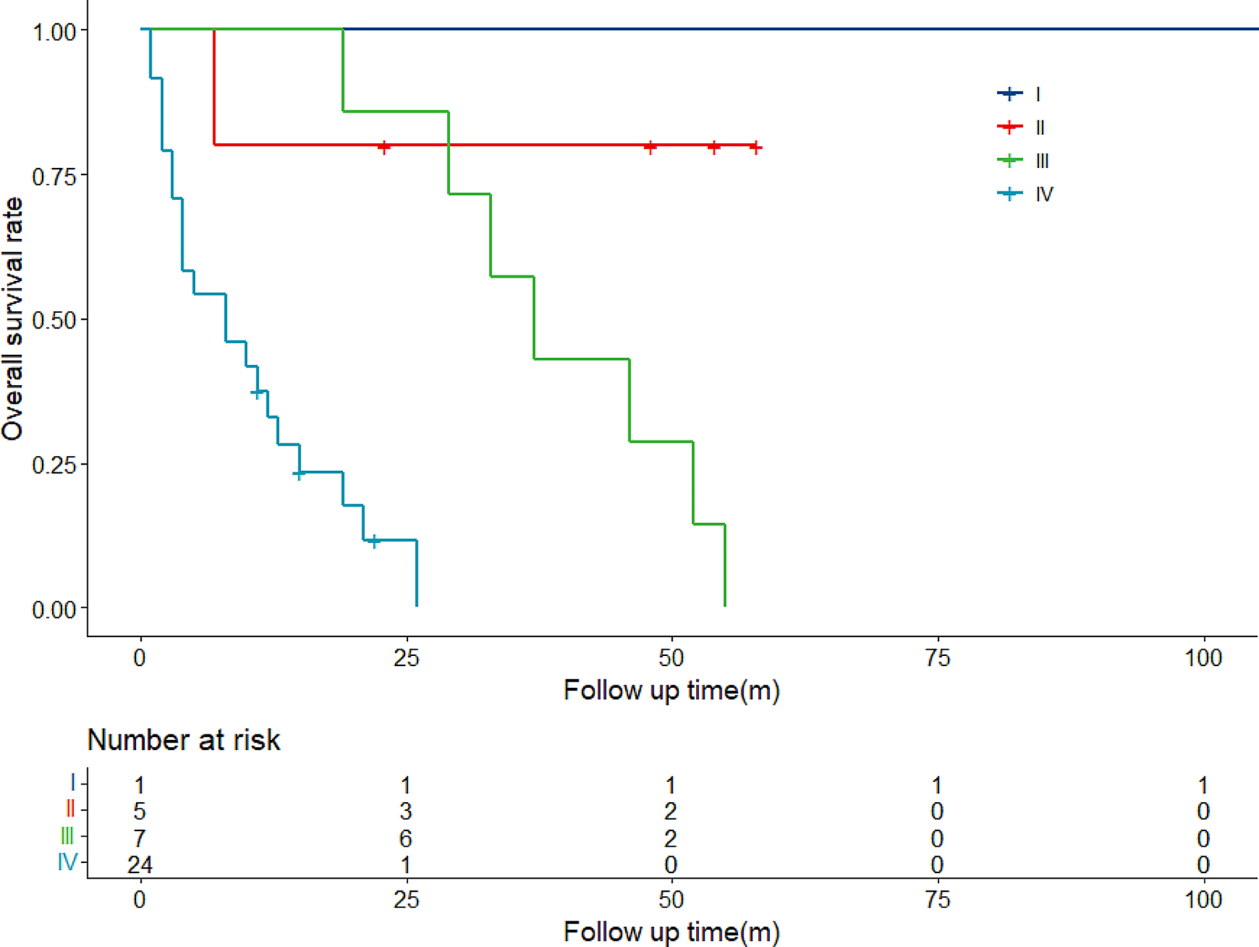
Overall survival rate of 37 GB-NEC patients in different stages

Survival analysis was performed according to different stages of disease, and the survival analysis of all patients is shown in Fig. 4. The median overall survival of patients with stages III and IV was 37 months (26.735–47.265) and 8 months (0.824–15.176), respectively, and the median overall survival time of patients with stage III was significantly longer than that of stage IV (37 months vs. 8.0 months, *P* < 0.001). Comparison of survival analysis in different stage of patients is shown in Table 6.

**Table 6:** Comparison of overall survival in different stage of patients

AJCC	I		II		III		IV	
	X^2^	*P*	X^2^	*P*	X^2^	*P*	X^2^	*P*
I			0.2	0.655	2.479	0.115	2.841	0.092
II	0.2	0.655			3.034	0.082	7.524	0.006
III	2.479	0.115	3.034	0.082			13.46	< 0.001
IV	2.841	0.092	7.524	0.006	13.46	< 0.001		

## Discussions

GB-NETs is a rare yet highly malignant gallbladder tumor, accounting for approximately 0.5% of all neuroendocrine neoplasms (NENs) in the body [9–12]. Typically, its pathological confirmation occurs after patients undergo excision surgery for cholecystitis or suspected gallbladder tumors. In contrast to gallbladder adenocarcinoma (GB-ADC), GB-NEC is often diagnosed at an advanced stage, marked by poor differentiation and frequent lymph node metastasis [13–15]. Research indicates a grim prognosis for GB-NEC, with a median survival ranging from approximately 3 to 14 months, and a majority of patients succumbing within 3 years of diagnosis [7, 14, 16]. The unfavorable prognosis is attributed to its heightened malignancy, strong invasiveness, and frequent identification in advanced stages [13, 17, 18]. However, Wang et al. observed that survival time in Stage I and II was significantly longer than that in Stage III and IV (*P* = 0.043), and the survival time of GB-NEC without liver metastasis exceeded that with liver metastasis (*P* = 0.013). This suggests that routine health check-ups may facilitate early diagnosis and treatment, potentially improving patients’ prognosis [19]. In our study, 24 cases (64.9%) were diagnosed at Stage IV, and 14 cases (37.8%) presented with distant metastasis, contributing to the observed poor prognosis.

In this study, we observed 37 cases of GB-NEC, encompassing 16 cases with concurrent cholecystolithiasis and cholecystitis, 3 cases of gallbladder polyps, 2 cases of gallbladder adenomyomatosis, and 1 case of common bile duct cyst. Additionally, there were 5 cases exhibiting the coexistence of NEC and gallbladder adenocarcinoma, with 2 cases meeting the diagnostic criteria for Mixed Neuroendocrine-Non-Neuroendocrine Neoplasms (MiNENs), while the remaining 3 cases had less than a 30% adenocarcinoma component. According to the 2019 WHO diagnostic criteria for MiNENs, these cases do not fully qualify as GB-MiNEN, but microscopic confirmation reveals the coexistence of two malignant tumor components [20]. The precise onset mechanism of GB-NEC remains a subject of debate. Some researchers propose that GB-NEC may originate from the metaplasia of gallbladder epithelial cells into gastric or intestinal epithelium. Prolonged chronic inflammatory stimulation is thought to promote the differentiation of epithelial cells toward neuroendocrine cells, offering a plausible explanation for the frequent association of GB-NEC with cholecystitis and cholelithiasis [6, 7, 21]. Conversely, other researchers contend that GB-NEC may derive from undifferentiated stem cells in the gallbladder or from gallbladder adenocarcinoma cells. As some GB-NECs are accompanied by adenocarcinoma, it has been confirmed that neuroendocrine carcinoma (NEC) and adenocarcinoma can undergo mutual transformation in gastrointestinal tumors [22, 23]. Additionally, neuroendocrine cells are scattered within the mucous glands at the neck of the gallbladder, suggesting that gallbladder neuroendocrine carcinoma (GB-NEC) may also originate from these cells [24]. Our research implies that the occurrence of concurrent biliary diseases in patients may be linked to dysplasia induced by prolonged chronic inflammation.

The incidence of GB-NEC is low, and it lacks specific clinical symptoms. Its radiological manifestations closely resemble those of adenocarcinoma, and laboratory tests do not offer biomarkers with both high sensitivity and strong specificity. Consequently, preoperative diagnosis is challenging, often resulting in misclassification as gallbladder adenocarcinoma. The gold standard for accurate diagnosis is pathological examination and immunohistochemical staining of resected or puncture biopsy specimens. Common biomarkers for GB-NEC immunohistochemistry include chromogranin A (CgA), synaptophysin (Syn), neuron-specific enolase (NSE), epithelial membrane antigen (EMA), and cytokeratins (CK). The first two biomarkers exhibit a relatively high positive rate in GB-NEC and are considered specific indicators for determining neuroendocrine differentiation in the tumor [25]. While the positive expression of neuroendocrine biomarkers aids in diagnosing neuroendocrine neoplasms (NEN), caution is warranted when CD56 is expressed alone [26]. Ki-67 serves to define the pathological grade, with a higher-level indicative of increased malignancy and a poorer prognosis. In this study encompassing 19 patients who underwent radical surgery, all 19 cases (100%) were positive for CgA, 12 cases (63.2%) were positive for Syn, and 16 cases (84.2%) exhibited Ki-67 levels exceeding 70%, consistent with previous research reports.

The study results revealed that patients with resectable lesions and surgical opportunities exhibited better prognoses compared to those with unresectable lesions. The treatment of gallbladder neuroendocrine carcinoma (GB-NEC) has been controversial, particularly the necessity of radical surgery. Some researchers express skepticism about the potential of surgery to enhance prognosis and extend survival. Kamboi M et al.‘s study reveals that a majority of GB-NEC cases are diagnosed in advanced stages, often with distant metastasis, limiting the option for surgery and leaving palliative anti-tumor treatment as the only alternative. Additionally, chemotherapy has shown promise in extending the survival of some patients [27]. Some studies suggest that the efficacy of surgical treatment is not significantly superior to chemotherapy. In our study, there was no significant difference in median survival between the surgical treatment group and the chemotherapy group (9.3 months and 8.0 months, respectively; *P* = 0.997) [28]. Contrary to the aforementioned perspective, most scholars assert that surgical resection is the preferred and primary treatment for GB-NECs. While a universally recognized surgical strategy is currently lacking, aggressive surgical resection is considered as an effective method to improve the survival rate and potentially the only cure for gallbladder tumors [11, 19, 21, 29]. Presently, the diagnosis and treatment of GB-NEC typically adhere to the diagnostic and treatment standards of gallbladder cancer (GBC) [9, 30]. Depending on the tumor’s staging, the surgical strategy and extent of resection vary. The specific range of surgical resection should be selected based on the tumor location, staging, and the patient’s overall condition, ranging from simple cholecystectomy to extensive curative or palliative resection [31]. Simple cholecystectomy is deemed feasible for localized T1N0M0 phase GB-NEC [6, 32, 33]. A study by Liu et al. [34] suggests that simple cholecystectomy suffices for T1N0M0 phase GB-NEC, with no adjuvant treatments postoperatively and no recurrence or metastasis during the 26-month follow-up period. However, for patients diagnosed in later stages without distant metastasis, radical tumor resection is the preferred treatment method. This involves removing the gallbladder and part of the liver, along with a corresponding regional lymph node dissection [35]. Radical resection surgery has been shown to prolong the survival time of patients with GB-NEC compared to other treatment methods [11, 36, 37]. A study based on the SEER database revealed that patients who underwent cholecystectomy had longer survival compared to those who did not undergo surgery. However, patients undergoing both cholecystectomy and lymph node dissection did not experience better survival benefits than those undergoing only cholecystectomy [10]. Some studies suggest that patients with clear N2 lymph node metastasis may not benefit from radical resection surgery, prompting intraoperative N2 lymph node biopsy and formulation of treatment regimens based on biopsy results [38–40]. The significance and value of surgery for patients with distant metastasis remain controversial. Some scholars argue that palliative tumor resection can reduce tumor burden, improve patients’ quality of life, and prolong survival [9, 41]. Research has also indicated that surgical procedures significantly improve overall postoperative survival compared to conservative treatment. This suggests that even in advanced patients, undergoing palliative tumor resection and postoperative comprehensive treatment can lead to survival benefits and provide favorable conditions for subsequent treatment [42]. In our study, although some patients lost the opportunity for curative resection at diagnosis, those who underwent surgical resection had a better prognosis compared to non-surgical treatment. Four patients underwent palliative tumor resection surgery, and 19 patients underwent curative resection treatment. The median survival times were 23 months and 19 months, respectively, which were significantly prolonged compared to patients receiving only chemotherapy (Group 1);In group 4, 7 (36.8%) patients were in stage IV at the time of diagnosis, whereas in group 1, 10 (83.3%) patients were in stage IV at the time of diagnosis, which may also be one of the factors influencing the difference in median survival time between the two groups. Therefore, for suspected GB-NEC patients, early diagnosis and efforts to complete radical resection are necessary, and active multimodal treatment may be a method to improve the survival rate [6].

Due to the rapid progression and high malignancy of GB-NEC, many patients are diagnosed in advanced stages, precluding the opportunity for curative surgery. Consequently, chemotherapy plays a crucial role in the treatment of such patients [8, 43]. The results of this study support the use of postoperative adjuvant therapy, demonstrating a significant extension in the median disease-free survival time and median overall survival time for patients who underwent radical surgery. Among the 19 patients included in the study, 11 received postoperative adjuvant therapy, while 8 did not. Compared to radical surgery resection alone, postoperative adjuvant therapy significantly prolonged the median progression-free survival time (5 months vs. 11 months, *p* = 0.041) and median overall survival time (13 months vs. 36 months, *p* = 0.032). Currently, the first-line chemotherapy regimens in China include EP (etoposide + cisplatin) and EC (etoposide + carboplatin), both of which have shown satisfactory clinical efficacy [44–46]. Previous studies have also supported the use of postoperative chemotherapy for GB-NEC patients. For example, Chu et al. [9] reported on seven cases of GB-NEC patients undergoing radical surgery, with three cases receiving postoperative EP regimen chemotherapy and four cases not receiving adjuvant therapy. They observed that postoperative chemotherapy extended the overall survival of patients by an average of 6.7 months compared to those who only underwent surgery. Similarly, in a retrospective study by Lee et al. [3], including 34 cases of GB-NEC, 22 patients received platinum-based chemotherapy postoperatively, three underwent postoperative radiotherapy, and nine did not receive any form of adjuvant treatment. Their analysis indicated that postoperative adjuvant therapy was an independent prognostic influencing factor (*P* = 0.007), significantly extending the median survival of the patients. Kim et al. [47] also reported a case of a GB-NEC patient with T3N1M0 who underwent laparoscopic radical cholecystectomy and postoperative combined radiochemotherapy. No evidence of recurrence was observed during a 14-month follow-up period. Thus, actively exploring postoperative adjuvant treatment strategies is essential to improving the postoperative survival rate after radical resection for GB-NEC.

With the rapid advancements in drug research and biological technology, targeted and immune therapies have demonstrated promising outcomes across various malignant tumors. However, there are no effective targeted drugs for GB-NEC. In a case study by Elahi et al. [48], a comprehensive therapeutic approach was employed for a GB-NEC patient. Following radical resection, the patient underwent gemcitabine and cisplatin chemotherapy. Upon recurrence with liver metastasis, a combination of docetaxel and sorafenib, along with hepatic radiofrequency ablation, was administered. The overall postoperative therapeutic response was favorable, resulting in a total survival time of 46 months. Liu et al. [49] reported a case of MiNENs patient (T3N2M0) who, due to a high PD-L1 positivity score, received six cycles of sintilimab monotherapy. After disease progression, the patient was treated with a combination of sintilimab and Tegafur for 8 cycles.The patient achieved a tumor-specific survival period of nearly 15 months. Up to the publication of the article, the patient has continued to receive the treatment regimen and maintained a good quality of life. Another noteworthy case, as reported by Chorath et al. [50] involved a patient with metastatic GB-NEC who exhibited a durable response to carboplatin, etoposide, nivolumab, and ipilimumab. This case suggests that incorporating checkpoint inhibitors alongside platinum-based chemotherapy may represent a promising and effective therapeutic option for GB-NEC.

Due to the endocrine-dependent biological behavior of NETs, the endocrine treatment is currently under intensive research. Somatostatin analogues like octreotide and lanreotide have inhibitory effects on tumor cell proliferation [51]. Research by some scholars revealed that octreotide can inhibit tumor progression, improve patients’ prognosis and alleviate tumor related symptoms [46, 52]. Therefore, for patients with positive expression of somatostatin receptors, somatostatin analogues, especially long-acting formulations, can be used as a new adjuvant therapy. Meanwhile, the use of radiolabeled somatostatin analogs for peptide receptor radionuclide therapy (PRRT) has also achieved good efficacy in the treatment of neuroendocrine tumors [53–59]. However, the majority of studies have focused only on NEN grade 1–2 (G1-2), and there is limited clinical research on NEN grade 3 (G3), which exhibit higher malignant biological behaviors [53, 54]. Nevertheless, it is gratifying that recent studies have confirmed the good efficacy and higher safety of PRRT in NEN G3 [55–59]. Zhang et al. reported a case of G3 pancreatic neuroendocrine neoplasms with liver metastasis treated with eight cycles of PRRT, resulting in a nearly decade-long survival with higher quality of life [57]. Similarly, Weich et al. reported a highly proliferative pancreatic neuroendocrine tumor (Ki67 = 60%) that received four cycles of PRRT after relapse following surgical treatment, leading to partial response [58]. Several single-center and multicenter studies on NEN G3 have shown that PRRT can achieve better efficacy than traditional chemotherapy and radiotherapy, resulting in longer progression-free and overall survival, while simultaneously with acceptable hematological and renal toxicities, as well as reliable safety [55, 56, 59]. Therefore, for NENs with high expression of somatostatin receptors and high proliferative activity, PRRT may be the most promising salvage treatment option.However, the majority of current research is based on small sample sizes, and the subjects are mainly derived from pancreatic neuroendocrine tumors.More research is needed in the future to explore the application of PRRT in GB-NEC.

In summary, GB-NEC presents as a rare and highly malignant tumor, primarily affecting middle-aged and older females. Pathological immunohistochemical examination remains pivotal for its confirmation, and surgical resection stands as the preferred treatment, significantly enhancing survival rates. Postoperative adjuvant chemotherapy, particularly platinum-based regimens, holds promise for improving outcomes in advanced cases. However, the efficacy of alternative treatments, such as immunotherapy and targeted therapy, requires further exploration.

Nonetheless, this study has several limitations. The extremely low incidence of GB-NEC and the constraints of single-center retrospective studies introduce challenges in addressing selection bias. Moreover, the absence of standardized adjuvant treatment protocols for GB-NEC may impact the research’s ability to ascertain its influence on survival rates. Moving forward, there is an urgent need for multicenter, larger-sample prospective studies to comprehensively analyze the clinical and pathological characteristics of GB-NEC, ultimately leading to the development of more tailored and effective treatment regimens.

## Data Availability

Availability of data and materials: All data generated or analyzed during this study are included in this published article (and its Supplementary Information files).
